# An Advanced Internet of Things System for Heatstroke Prevention with a Noninvasive Dual-Heat-Flux Thermometer

**DOI:** 10.3390/s22249985

**Published:** 2022-12-18

**Authors:** Toshiyo Tamura, Ming Huang, Takumi Yoshimura, Shinjiro Umezu, Toru Ogata

**Affiliations:** 1 Institute of Healthcare Robotic, Future Robotics Organization, Waseda University, Tokyo 169-8050, Japan; 2 Computational Systems Biology, Division of Information Science, Nara Institute of Science and Technology, Ikoma 630-0192, Japan; 3 Medical and Welfare Engineering Program, Tokyo Metropolitan College of Industrial Technology, Tokyo 116-8523, Japan; 4 Department of Modern Mechanical Engineering, Faculty of Science and Engineering, Waseda University, Tokyo 169-8555, Japan; 5 Rehabilitation Medicine, School of Medicine, University of Tokyo, Tokyo 113-0033, Japan

**Keywords:** heatstroke, deep body temperature, dual-heat-flux thermometer, thermal regulation, IoT system, wearable device, continuous non-invasive temperature monitoring, core temperature, clinical thermometry

## Abstract

Heatstroke is a concern during sudden heat waves. We designed and prototyped an Internet of Things system for heatstroke prevention, which integrates physiological information, including deep body temperature (DBT), based on the dual-heat-flux method. A dual-heat-flux thermometer developed to monitor DBT in real-time was also evaluated. Real-time readings from the thermometer are stored on a cloud platform and processed by a decision rule, which can alert the user to heatstroke. Although the validation of the system is ongoing, its feasibility is demonstrated in a preliminary experiment.

## 1. Introduction

Heatstroke is caused by overheating, typically as a result of prolonged physical exertion at high temperatures [1,2]. As the most serious type of heat injury, heatstroke occurs if the body temperature rises to 40 °C. Heatstroke often occurs when there is a sudden change in ambient temperature (e.g., from spring to summer) and the thermoregulation system is not functioning optimally. The risk of heatstroke at high temperatures differs according to age. Heatstroke requires emergency treatment. Untreated heatstroke can cause permanent damage to the brain, heart, kidneys, and muscles. Continuous monitoring of vital signs can provide physiological information, and a digital marker sensitive to subtle physiological changes may be useful for disease prevention. A shift in vital-sign measurement from a hospital to an ambulatory setting has been facilitated by the popularity of wearable devices.

The symptoms of heatstroke include high body temperature, nausea, vomiting, flushed skin, high heart rate, and rapid breathing. Some symptoms can be monitored by wearable devices combined with biosensors. Deep body temperature (DBT) is difficult to measure noninvasively given that it reflects the temperature within body cavities; therefore, invasive measurement is necessary, typically of the rectum temperature in nosocomial settings. DBT monitoring is needed not only in hospitals but also in daily healthcare. The real-time physiological monitoring of construction workers is important because there are strict regulations to protect outdoor workers. For this purpose, ambient temperature control based on the wet bulb global temperature (WBGT) is currently used [3]. However, given the interindividual differences in thermal regularization, an individual index is needed. Wearable devices enable the collection of physiological information about the user. Heat strain control is a crucial measure, especially in summer. In Japan, it is recommended that workers wear long-sleeve jacket and trousers for safety reasons. However, this hampers heat dissipation. Hence, close monitoring is needed.

Thermal regulation requires the monitoring of DBT rather than skin temperature. Strictly speaking, only noninvasive methods are desirable for DBT measurement. Some medical devices such as the CoreTemp (TERUMO Co., Tokyo, Japan) and Bair Hugger (3M, Saint Paul. MN, USA) [4,5,6,7,8] can accurately approximate the DBT. These devices are fabricated based on the zero-heat-flux method (ZHFM) [4], which requires an external power supply to achieve heat equilibrium in the probe. Devices based on ZHFM are active sensors, which typically contain a self-heating layer to prevent heat transfer between the skin and external environment, which makes the probe temperature equivalent to the DBT [5]. A servo-controlled heater that requires considerable power is necessary to compensate for heat loss to the ambient environment. Given the power consumption and risk of low-temperature burns, prolonged use is difficult. Newer sensors with no heater can accurately approximate DBT, for example, the aural canal thermistor (ACT) measures the temperature of the tympanic membrane [9]. The DBT thermometer based on the dual-heat-flux method [10,11,12] is wearable and suitable for prolonged use. In contrast to the ZHFM, the dual-heat-flux method (DHFM) (Section 3) estimates/calculates the DBT using two heat fluxes from the core through the skin into the probe. To calculate the DBT, the two heat fluxes must travel via two paths with different thermal resistances. Compared with the ACT, which is used only at rest because it is inserted into the ear canal, the DHFM is theoretically capable of being used for monitoring devises without requiring the subject to be at rest.

In the past, heatstroke-prevention systems based on surface temperature (ST) and heart-rate measurements have been developed [13,14,15]. DBT has been predicted using algorithms such as the fuzzy control [13], conversion [14], and bioheat transfer equations [15]. However, none of these studies attempted to measure DBT directly.

In this study, we developed an Internet of Things (IoT) system consisting of an improved wearable DHFM-based thermometer with high accuracy and a DBT prediction model based on sensor reading. We validated the system in a high-temperature environment. The feasibility and performance of the system are discussed further in [2,3,4,5,6]. See the end of the document for further details on references.

## 2. System Concept

**Figure 1:**
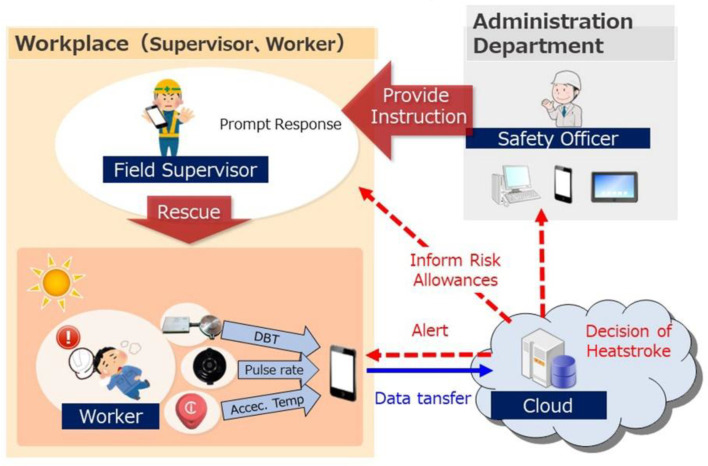
The IoT heatstroke-prevention system.

DBT, which reflects the core temperature of a body cavity, provides physiological information, indicates immunologic functional status, and reflects the circadian rhythm. DBT is more stable than skin temperature because it is less affected by the ambient environment. We developed a rapid and easy-to-use IoT system for heatstroke prevention (Figure 1).

The DBT, heart rate, and acceleration are measured, and ambient temperature and humidity are obtained as references. These physiological and environmental data are used to predict the risk of heatstroke and prompt the user to take preventive measures as necessary. Low-power Bluetooth wireless communication is used to transmit data to a smartphone. Despite claims of a transmission range of ~ 100 m, Bluetooth devices reliability operate at a range of 5–10 m. This may be insufficient for monitoring the physical status of an outdoor worker. Therefore, we added a microSD memory card to prevent loss of data.

The heatstroke risk level is calculated using physiological and environmental information collected by the sensor, and the user is warned to check their physical state to prevent thermal damage. The device was designed to maximize ease of use.

## 3. Wearable Deep Body Thermometer

Wearable devices can automatically and instantly monitor physiological information. Heatstroke warnings from wearable devices will be useful for users in hazardous environments or with poor health, by making them aware of the risk of heatstroke. Therefore, we designed a wearable heatstroke detection device for workers using a deep body thermometer.

### 3.1. Principle of the Dual-Heat-Flux Thermometer

**Figure 2:**
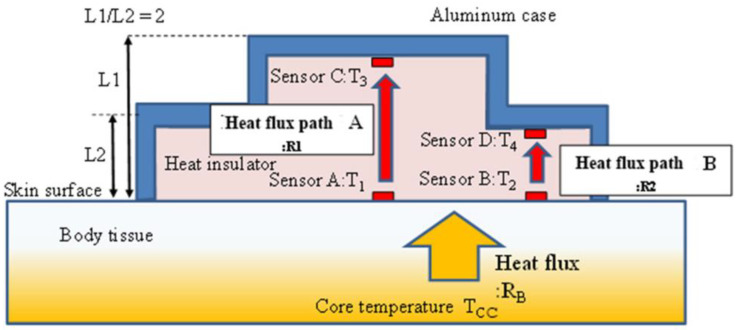
Principle of the dual-heat-flux thermometer.

A dual-heat-flux thermometer (DHFT) calculates DBT based on the heat flux inside a probe and was proposed by Kitamura et al. [9]. By doubling the heat path inside the probe, the DBT can be calculated by embedded temperature sensors. The principle of DHFT is shown in Figure 2. A substrate material with four embedded temperature sensors is the core of the probe. The substrate material has similar physical properties to skin; when attached to skin, heat from the core body arising from the difference between the DBT and skin temperature flows into the substrate material. Additionally, through a “heat isolation peripheral boundary condition”, heat flows longitudinally. Because the two heat paths (*T*_1_ − *T*_3_ and *T*_2_ − T_4_) are transversely proximal, the thermal resistors in the skin layer of the two heat paths are identical. Thus, DBT can be calculated by the four sensors using the equation below, where *k* (=R1/R2 in Figure 2) is the ratio of the probe heat resistors in the two heat paths [9].

(1) $$T_{d}=T_{1}+\frac{\left(T_{1}-T_{2}\right)\left(T_{1}-T_{3}\right)}{k\left(T_{2}-T_{4}\right)-\left(T_{1}-T_{3}\right)}$$

The prototype had an accuracy of <0.1 °C relative to the reference deep body thermometer, although an additional urethane sponge cover must be used. The method was modified by Huang et al. based on theoretical simulations and experimental validation [10,11] (Equation (2)). The first assumption is that heat flows vertically to the surface. However, as shown by using finite element simulations, the heat flew into the probe in a diffuse manner. Accuracy can be improved by modifying the conventional formula:

(2) $$T_{\mathrm{cc}}=T_{1}+\frac{\left(T_{1}-T_{2}\right)\left(T_{1}-T_{3}\right)}{k\left(T_{2}-T_{4}\right)-\left(T_{1}-T_{2}\right)},\;k=2$$

This method is more suited to long-term measurements than the zero-heat flow method, which uses a heater because of the low power consumption of that device. The absence of an external heater markedly reduces energy consumption, which facilitates wearable applications.

### 3.2. The Effect of Ambient Temperature and Calibration Device

The deep body thermometer is used to monitor core temperature noninvasively during surgery, and for patients in critical condition in intensive care, where ambient temperature is constantly maintained. By contrast, a wearable thermometer monitors DBT in daily life, so the DHFT must consider the effect of ambient temperature.

**Figure 3:**
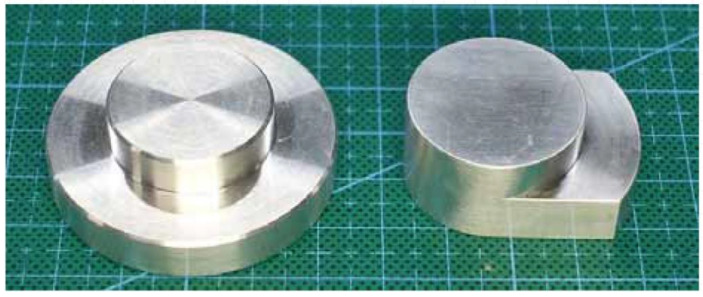
Prototype probes.

We designed two types of probes (Figure 3). The probe on the left is the original type (40 mm in diameter), and that on the right is a miniature probe with a length of 30 mm.

**Figure 4:**
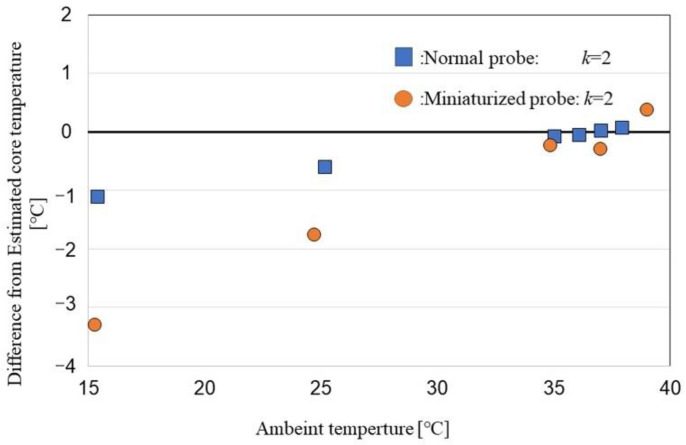
DBT error according to ambient temperature.

The effect of ambient temperature is shown in Figure 4. Accuracy is affected by ambient temperature; error increases as ambient temperature decreases, possibly because total thermal resistances combined with probe material (aluminum) and heat-insulator at different ambient temperatures.

**Figure 5:**
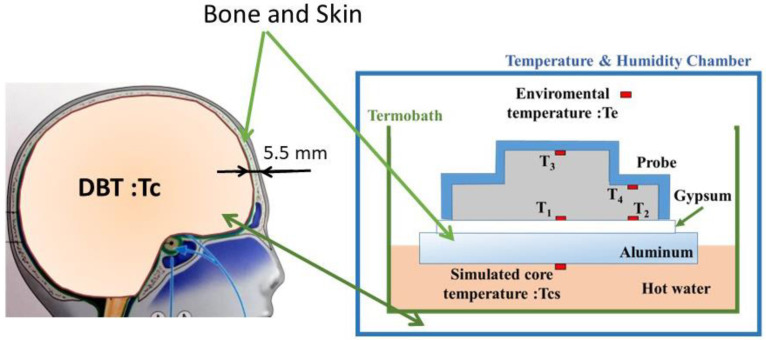
Calibration device mimicking the forehead.

The thermal resistance ratio, *k*, is defined as the ratio of the probe heights. We constructed a calibration system consisting of a thermostatic water bath to simulate brain temperature, and a layer of gypsum with thermal conductivity similar to the forehead (bone and skin) (Table 1, Figure 5).

Due to the temperature difference, heat flows through the aluminum plate and gypsum layer into the probe. Because aluminum has high thermal conductivity, the delay of the heat flow can be ignored. The experiment was conducted in a thermal chamber with the temperature controlled to within ±0.05 °C. We simulated ambient temperatures of 15–36 °C at a constant core temperature (water temperature) of 37°C. The ambient temperature was measured at air inside the chamber. Then, estimated the DBT Tcs is as follows:

(3) $$T_{\mathrm{cs}}=T_{1}+\frac{\left(T_{1}-T_{2}\right)\left(T_{1}-T_{3}\right)}{k\left(T_{2}-T_{4}\right)-\left(T_{1}-T_{2}\right)}$$

where *T*_1_, *T*_2_, *T*_3,_ and *T*_4_ are the measured temperature values. It follows that

(4) $$k=\frac{\left(T_{cs}-T_{3}\right)\left(T_{1}-T_{2}\right)}{\left(T_{cs}-T_{1}\right)\left(T_{2}-T_{4}\right)}$$

*k_tei_* at different ambient temperatures, *T*_ai_, is obtained, followed by the averaged *k* value *k_av_*):

(5) $$k_{av}=\frac{1}{n}\overset{n}{\underset{Tai=1}{\sum }}k_{tei}$$

### 3.3. Evaluation Study

With using of known core temperature Tcs. For different ambient temperatures, different k values are obtained, and at the end an average value = *k_av_* are calculated by Equation (5).

**Figure 6:**
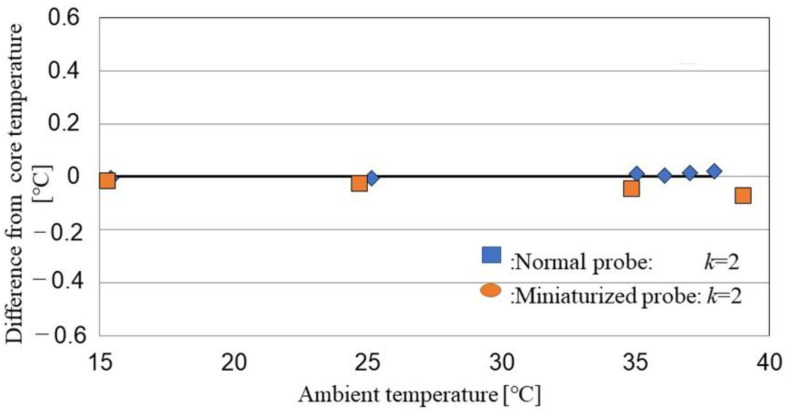
DBT error according to ambient temperature under the calibration system.

The calculated *k_av_*-values of the standard and miniature probes at different ambient temperatures were 1.42 ± 0.01 and 0.98 ± 0.03 (mean ± standard error), respectively (Figure 6). The estimated error was −0.46 ± 0.50 at a constant *k* ratio of 2. Therefore, thermal resistance is affected by ambient temperature, and a calibrated k-value should be used to minimize the error in DBT measurement.

This result indicated thermal gradient affected by the ambient temperature.

## 4. Prediction Model

Thermal modeling is a quantifiable and repeatable method of predicting thermal and physiological responses to various conditions that enables data-driven guidance. Early models were designed to address specific environmental conditions. However, the models have become increasingly more sophisticated to provide higher-resolution information on human physiological responses, and the use of combinations of models has become necessary. Although the validation of thermoregulation models is important for increasing confidence in their results, their utility for monitoring physiological parameters is limited. In the real world, the number of monitoring devices must be controlled. A small number of environmental and physiological variables, as well as physical activity and clothing properties, are used to simplify the heat balance equation. In prior studies of core-temperature estimation, only skin temperature, skin heat flux, and heart rate were used to estimate core temperature using a Kalman filter [16] and a linear regression model [17] due to the lack of core temperature information. In our model, however, the temperature was directly detected by the DHFT, and predictions were made based on DBT changes.

**Figure 7:**
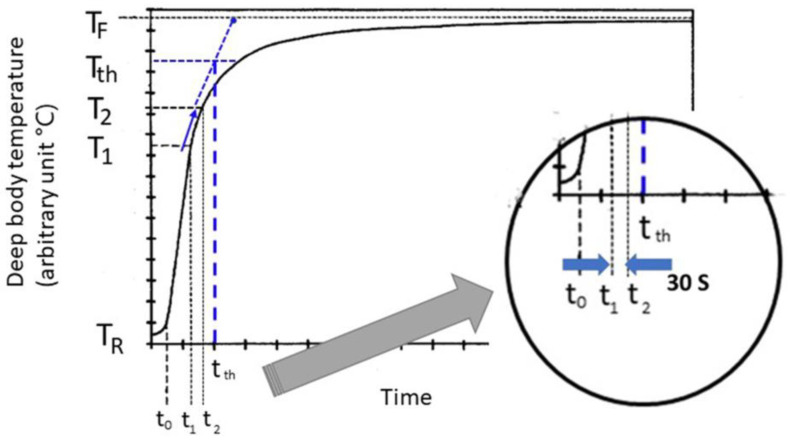
Sensor temperature signals over time showing the measurement start time, a critical measurement interval, and the stabilization temperature during exercise. Signals calculated with 60 s moving averages and estimation were made by temperature changes with 30 s.

Figure 7 shows typical temperature changes during exercise. The temperature indicated by the device lags behind the actual temperature T of the subject. Therefore, beginning at t_0_, the temperature rapidly increases from T_R_ to T_1_ at times t_0_ and t_1_. The rate of increase in the indicated temperature is relatively stable between t_1_ and t_2_, and thereafter it gradually returns to the stabilization temperature T_F_. Our system is capable of analyzing early temperature data, for example, between t_1_ and t_2_, and predicting the final temperature, T_F_.

There is a need, therefore, for a measurement system predictive of a stabilization temperature that can adapt to the changing heat-flow characteristics of both the body under measurement and the measurement system itself, unlike a first-order model. Adaptive techniques that use sets of simultaneous equations solved in real-time to yield a likely temperature-rise curve that indicates the stabilization temperature have been proposed. However, a considerable amount of time may be required to acquire the temperature-rise curve. The goal is to predict the stabilization temperature as early as possible.

The temperature curve has a parabolic shape during the initial temperature rise (Figure 7). During this parabolic phase, the sustained slope changes between T_1_ and T_2_ predict the final stabilized temperature. t_1_ is defined as a temperature difference of >0.1 °C at a moving average of 60 s. After 30 s, t_2_ is detected and the slope is calculated. The relationship between T_1_ and T_2_ is assumed to be a liner fashion and the slope (dT/dt) is simply defined as

(6) $$\mathrm{Slope}=(T_{2}-T_{1})/(t_{2}-t_{1})$$

The threshold temperature T_th_ is determined as 95% of a new steady state temperature T_F_, e.g., 0.95 (T_F_ − T_R_) + T_R_, and the crossing point time t_th_ between an extended line of slope and T_th_ is calculated. If the real deep body temperature is above the threshold temperature at time t_th_, a warning is transmitted to the worker. In our evaluation, T_F_ was determined at 38 °C.

## 5. Experiment

### Experimental Protocol

We evaluated the performance and feasibility of the wearable thermometers for the prevention of heatstroke in a high-temperature environment with a physical workload using the zero-heat flow thermometer (ZHFT) and the DHFT. In addition, the STs inside and outside the jacket were monitored to determine the user’s thermal status.

Eight young male subjects (age, 25.3 ± 8.9 years; height, 169.6 ± 5.2 cm; and weight, 64.7 ± 8.6 kg) participated in the experiments. The experiment was conducted in an isothermal chamber with an ambient temperature of 39 °C and 40% relative humidity. Each experiment comprised acclimation (10 min), cycling exercise (20 min, 50 W), and recovery (10 min) phases.

**Figure 8:**
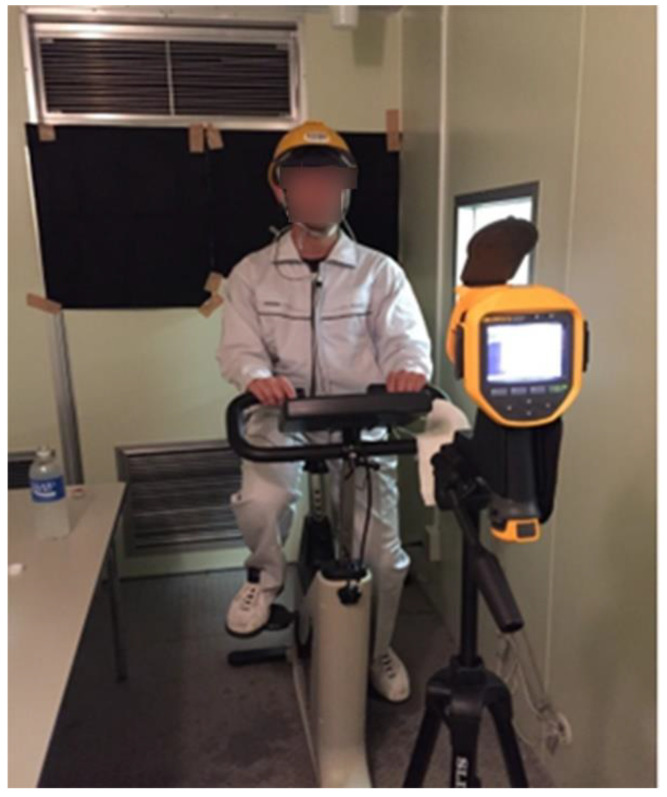
Experiment setup.

DBTs on the forehead and the STs inside and outside the jacket were measured using a ZHFT (CoreTemp CM-210; TERUMO Co, Tokyo, Japan), the prototype DHFT, and a skin thermometer (N543RV; Nikkiso-Thermo Co., Ltd., Tokyo, Japan). Both DBTs recorded the temperature at 2-s intervals. The ZHFT and DHFT were applied to the forehead beneath a helmet and sampled at 1-s intervals (Figure 8).

We evaluated the availability of DHFT in an environment with a higher temperature than the DBT. Because the ZHFT has a maximum working temperature of 40 °C with a 0.1 °C error, it was used as the reference. We also evaluated the use of ST as the only indicator of thermal status.

The experiments were approved by the Ethics Committees of Tokyo Metropolitan College of Industrial Engineering and were conducted in collaboration with Waseda University and Nara Institute of Science and Technology (approved code 3-ITArakawa578 and date of approval 18 November 2021). The subjects provided informed consent to participate in the experiment.

## 6. Results

Because the DHFT is a passive device, the time lag exceeds (279)(283) 5 min depending on the ambient temperature. Thus, the temperature was sufficiently stable during the acclimation stage. The ST and DBT measured by the skin thermometer and DHFT are listed in Table 2. The paired *t*-test was used to assess the significance of differences between the exercise and recovery phases; the acclimation phase data were not analyzed. There was no significant difference between ST and DBT (Table 2).

**Figure 9:**
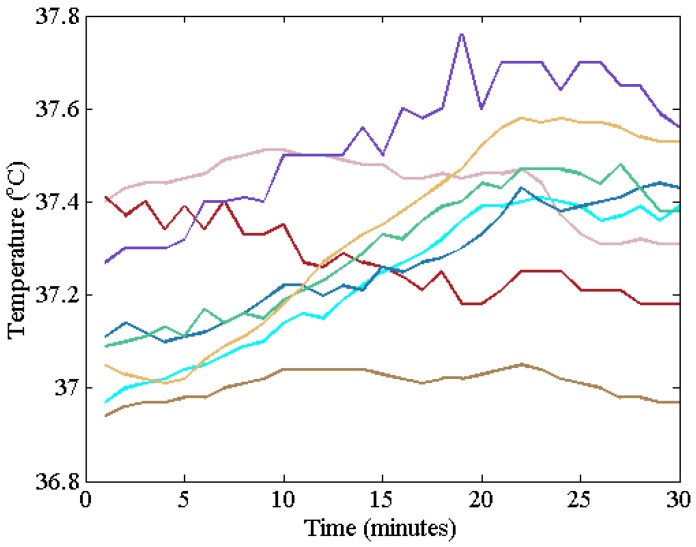
DBT measured on the forehead of eight subjects (each color represents an individual).

Figure 9 shows forehead temperatures measured by the DHFT. Although the ambient temperature was set at 39 °C and the ST was above 38 °C, the core temperature was below 38 °C. Several parabolic shapes were observed. The predetermined threshold temperature was 38 °C, so no warning was transmitted in this experiment.

**Figure 10:**
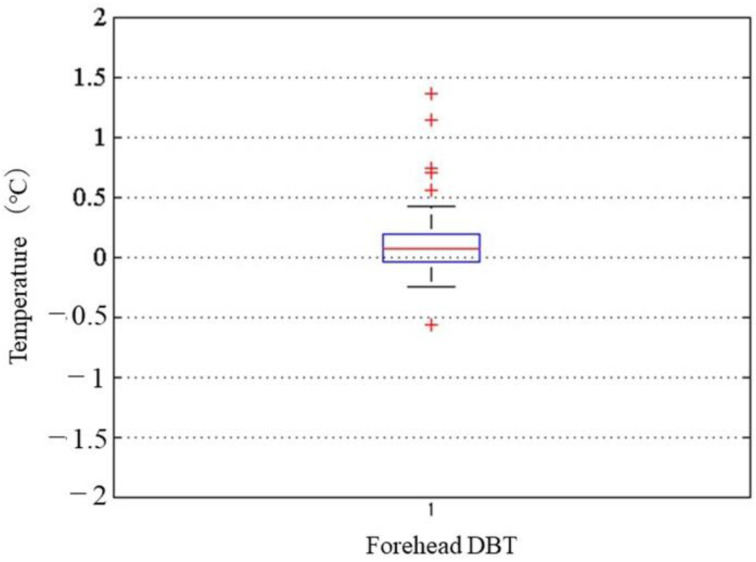
Box plot of DBT during the exercise and recovery periods. DBT measured on the forehead by the ZHFT and DHFT.

**Figure 11:**
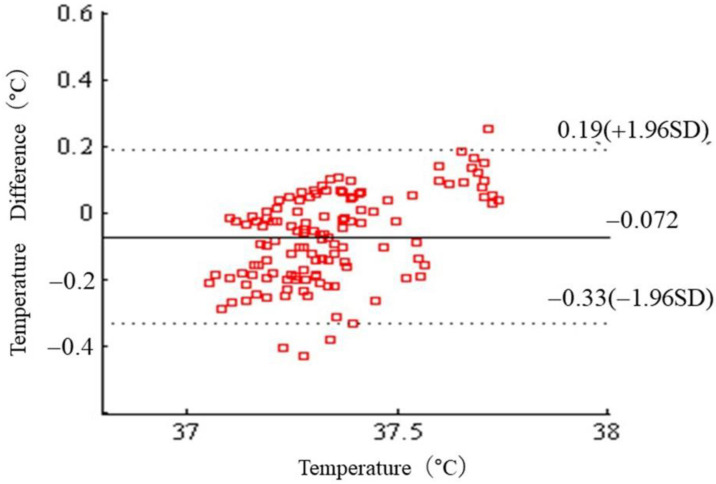
Bland–Altman plot of DBT during the exercise and recovery periods. Difference between ZHFT and DHFT measurements of DBT on the forehead.

In the box plot, outliers were considered abnormal measurements (caused by the sensor peeling off or by sweat) (Figure 10). We then generated a Bland–Altman plot (Figure 11). The average difference between the ZHFT and DHFT was 0.07 ± 0.33 °C (95% CI −0.3 to 0.2). 

In the evaluation of the model, the body temperature in our study did not exceed 38 °C. The threshold temperatures of individuals were calculated between 39.93 °C and 39.97 °C. None of the subjects exceeded the threshold temperature.

## 7. Discussion

### 7.1. Dual-Heat-Flux Thermometer

The desire to monitor health data is driving the rapid growth of wearable technologies to measure body temperature, among other physiological parameters. The accurate and continuous measurement of DBT by a wearable device is important for healthcare and disease monitoring. However, the accurate monitoring of DBT is challenging; therefore, accurate and continuous DBT thermometers are not readily available. The DHFT has shown promise [18,19,20] and thus was developed herein; high accuracy was achieved.

We developed a heatstroke-prevention system based on a deep body thermometer. This is the first report of direct measurement of DBT using a noninvasive body thermometer. The DHFT is a passive device and must be periodically stabilized. The temperature is sensitive to changes in activity after the DHFT has achieved equilibrium. Monitoring temperature trends in DHFT provides information on core body temperature, which is useful for assessing heat stress.

This study provides data on the accuracy of a non-invasive heat-flux-based thermometer for continuous monitoring of core body temperature. The estimated core temperatures showed good agreement (0.07 ± 0.33 °C) and no proportional bias relative to the clinically approved deep body thermometer (CoreTemp), which is commonly used in operating theaters. With precise calibration, its accuracy complies with the ISO 80601-2-56 standard (≤±0.2 °C) and the Japanese industrial standard JIST1140 (≤±0.1 °C). The mean error was comparable to the ZHFT temperature measurements, with a calculated limit of agreement within ± 0.1 °C.

Although the developed thermometer has limitations for assessing core body temperature during exercise, our system might be useful for detecting hyperthermic events. Our novel non-invasive DHFT was able to detect changes in body temperature.

### 7.2. Prediction of Heatstroke

The results indicated that the core body temperature did not exceed 38 °C during the experiments. Although the DHFT aids the assessment of the relationship between behavior and physiological effects, further studies on its usability are required. In this study, the thermometer was attached to a helmet. Although miniaturized, it can cause discomfort if used over the long term; small and precise thermometers are thus needed.

The prediction algorithm was not fully evaluated in our experiments, but the predetermined value allowed for safe and continuous working.

Most prior studies used thermoregulation models based on skin temperature and heart rate. Multiple parameters are typically included in core-temperature prediction equations [3,16,17,21,22,23,24,25]. However, in real working conditions, monitoring systems using simple assumptions are preferred for continuous evaluation.

### 7.3. Proposed System

Our proposed device (Figure 1) measures DBT, heart rate, acceleration, ambient temperature, and humidity. In this study, we focused on DBT measurement, although heart rate actually changes more rapidly than DBT during exercise. Therefore, heart rate and pulse rate measurements may be useful parameters for heatstroke-prevention systems [16,17,21,22].

The multiple-parameter approach renders our device useful for personal healthcare, including the determination of individual risk to high temperatures. Our system has a compact design and is inexpensive. Compact designs improve the comfort and utility of health-monitoring systems. Compared to other available systems, our device is easily adaptable for comfortable use.

Low-cost options for personalized health monitoring are of particular interest to vulnerable populations, such as the elderly, who may not have access to telehealth. For elderly persons in institutions or living alone at home, such monitoring systems may be of particular benefit, and personal wearables for health monitoring can meet critical healthcare needs. Additionally, such technology may facilitate research on the effects of hot environments on the body. The emergence of health informatics and telehealth necessitates low-cost, comfortable, and unobtrusive systems that can be seamlessly integrated into daily life.

## 8. Conclusions

In conclusion, a heatstroke-prevention system with a non-invasive dual-heat-flux thermometer is a feasible alternative to a ZHFM for assessing core temperature of outdoor workers. The core temperatures estimated by the DHFM showed good agreement with those of the ZHFM. A simple prevention algorithm was proposed but not evaluated; further studies are therefore needed.

## Figures and Tables

**Table 1:** Thermal conductivity of different materials.

Material	Thermal Conductivity (W/m K)
Skin	0.45
Bone	0.45
Gypsum	0.43

**Table 2:** Temperatures during the exercise and recovery phases.

Temperature	During Exercise	During Recovery
Surface (outside jacket)	38.96 ± 0.23	38.94 ± 0.13
Surface (inside jacket)	38.36 ± 0.19	38.39 ± 0.12
Forehead DHFT	37.26 ± 0.09	37.38 ± 0.03

## Data Availability

Data of our study are available upon request.
